# A case of slowly progressive malignant pericardial mesothelioma suggesting the involvement of BAP1 loss

**DOI:** 10.1002/rcr2.1004

**Published:** 2022-08-06

**Authors:** Naoto Fukasawa, Yoko Agemi, Aya Shiba, Masaharu Aga, Yusuke Hamakawa, Kazuhito Miyazaki, Yuri Taniguchi, Yuki Misumi, Tsuneo Shimokawa, Kyoko Ono, Hiroyuki Hayashi, Hiroaki Okamoto

**Affiliations:** ^1^ Department of Respiratory Medicine Yokohama Municipal Citizen's Hospital Yokohama Japan; ^2^ Department of Pathology Kanagawa Cancer Center Yokohama Japan; ^3^ Department of Pathology Yokohama Municipal Citizen's Hospital Yokohama Japan

**Keywords:** BAP1, cell block, long‐term survival, malignant pericardial mesothelioma, pericardial effusion

## Abstract

Malignant pericardial mesothelioma (MPM) is a rare tumour that arises from the mesothelial cells of the pericardium. No standard treatment has been established owing to a poor treatment response; therefore, MPM has a poor prognosis. We herein report a rare case of MPM in a 70‐year‐old man that was diagnosed immunohistopathologically using cell block sections of pericardial fluid and in which long‐term survival for more than 3 years was achieved with only periodic pericardial drainage. Immunohistopathological staining investigations, especially BRCA1‐associated protein 1 (BAP1) immunostaining using cell block sections of pericardial effusion, are effective in making a diagnosis of MPM. Well‐differentiated papillary mesothelioma (WDPM) with BAP1 loss progresses to MPM in the long term, showing that BAP1 loss may induce phenotypical evolution of WDPM. BAP1 loss may also progress to malignant mesothelioma in situ and then to invasive mesothelioma. BAP1 immunohistochemistry should be considered for the early diagnosis of MPM.

## Graphical abstract

We report a rare case of malignant pericardial mesothelioma diagnosed immunohistopathologically using cell block sections of pericardial fluid and the patient had prolonged survival for more than 3 years with only periodic pericardial drainage.

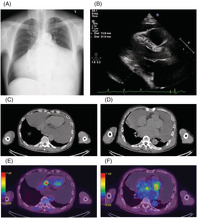

## INTRODUCTION

Malignant pericardial mesothelioma (MPM) is a rare tumour derived from pericardial mesothelial cells and accounts for only 0.7% of all mesothelioma cases.^1^ Immunostaining investigations using cell block sections of the pericardial effusion are effective for making the diagnosis of MPM when pericardial biopsy is difficult. MPM has a poor prognosis, with a median survival of less than 6 months from presentation.^2^ Herein, we report a rare case of MPM diagnosed immunohistopathologically using cell block sections of pericardial fluid and the patient had prolonged survival for more than 3 years with only periodic pericardial drainage.

## CASE REPORT

A 70‐year‐old man with a medical history of atrial fibrillation, chronic kidney disease, chronic heart failure and hypertension presented to his previous doctor with complaints of tightness of the chest. Chest radiography performed 2 years ago revealed cardiac enlargement. He had smoked 1 pack per day for 25 years until the age of 45 years. He had worked at a press shop for 3 years from the age of 15 and may have had low‐level exposure to asbestos. He was found to have significant pericardial effusion, and pericardial drainage was therefore performed; however, cytology findings showed class IIIb and did not reveal the cause of the pericardial effusion. His symptoms temporarily improved with pericardial drainage, but 2 weeks later, pericardial effusion was observed at the same volume as that before drainage. Over the next 2 years, he underwent repetitive drainage every 3 months and was referred to our department for further examination and treatment.

On physical examination, he had no abnormalities, including symptoms of heart failure. Laboratory data showed the following findings: blood urea nitrogen, 29.1 mg/dl; creatinine, 1.62 mg/dl; brain natriuretic peptide, 103.7 pg/ml. Serum tumour markers for lung carcinoma showed a negative result.

**FIGURE 1:**
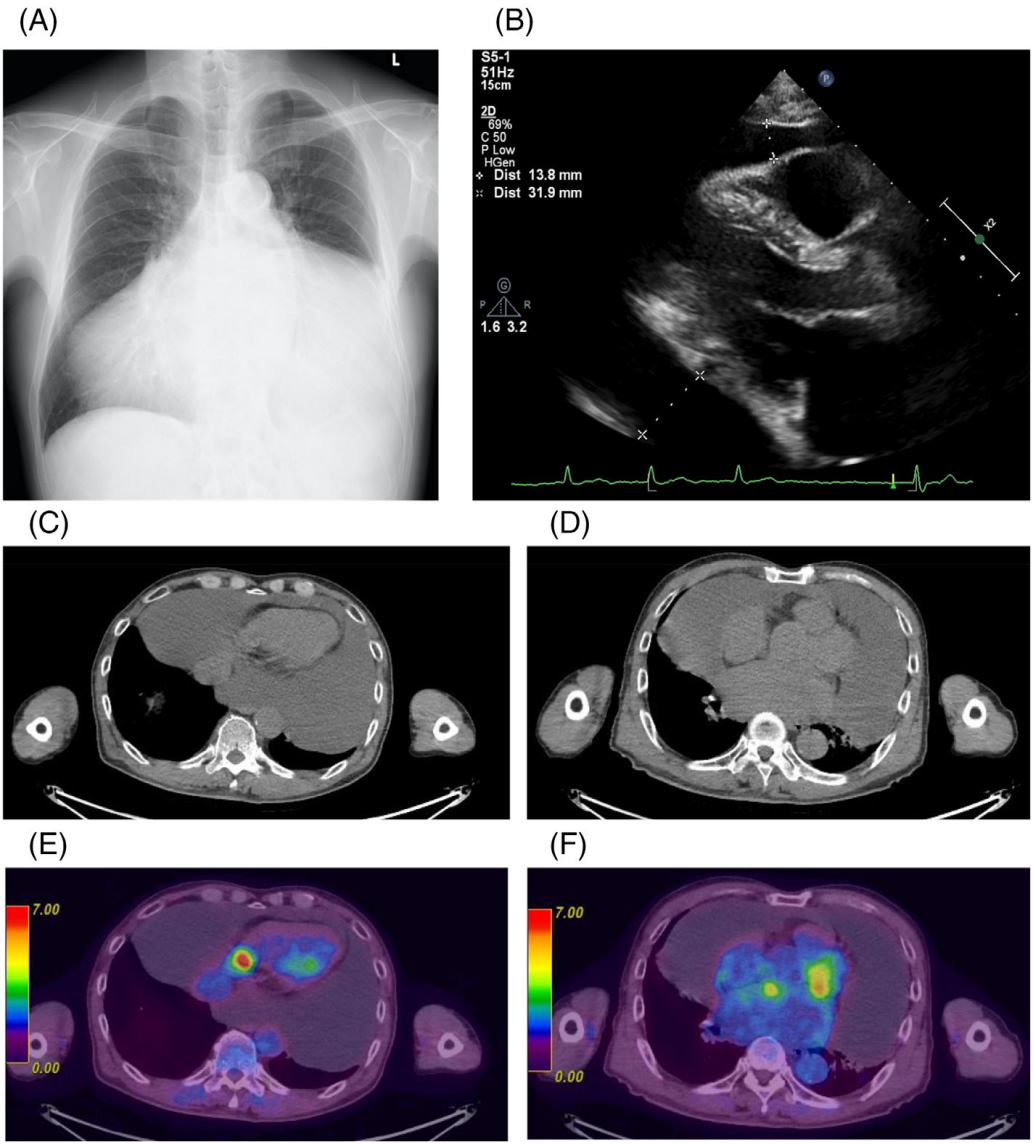
Chest radiography (A) and percutaneous echocardiography (B) taken at initial presentation. Notable cardiac enlargement and massive pericardial effusion are observed. Chest computed tomography (CT) (C, D) and ^18^F‐fluorodeoxyglucose (FDG) positron emission tomography‐CT (E, F) scans at initial presentation. The image shows increased uptake of FDG on the right ventricular caudal side (C and E), around the pulmonary artery and around the root of the aorta (D and F).

**FIGURE 2:**
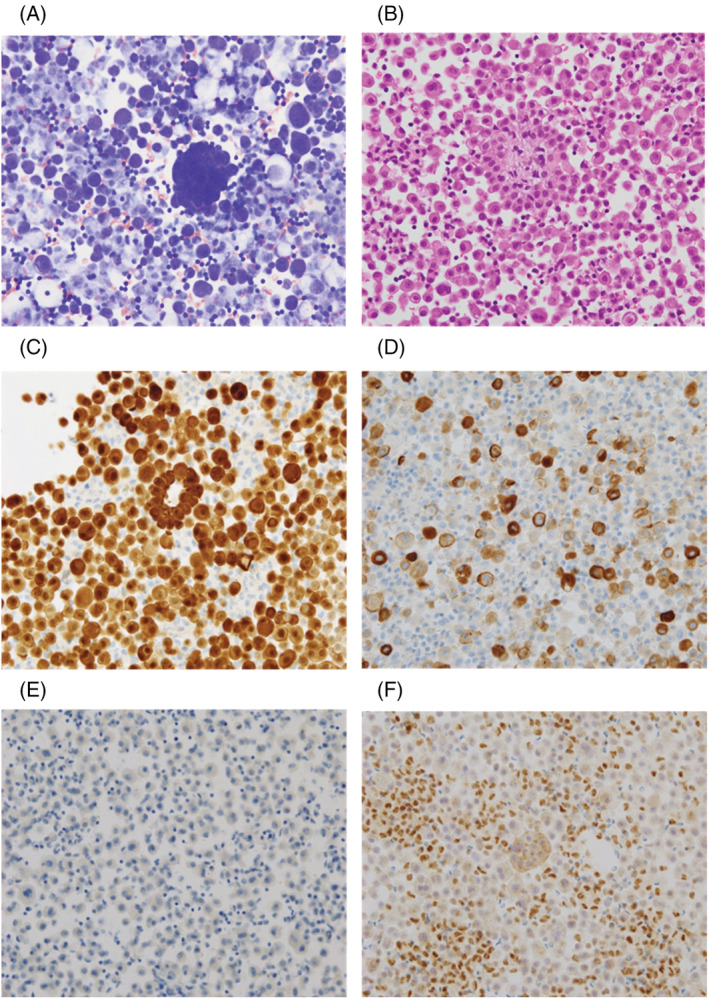
Cytology of the patient's pericardial fluid. Giemsa staining images (A, ×200). Mesothelial cells with minimal atypia are diffusely observed in the image. It is difficult to differentiate mesothelial cells from reactive benign mesothelial cells. Cell block sections show high cellularity composed of atypical cells with enlarged nuclei and eosinophilic cytoplasm including papillary structures. Haematoxylin–eosin staining images (B, ×200). The cells were diffusely positive for calretinin (C, ×200), focally positive for EMA (D, ×200) and negative for CEA (E, ×200), together with the loss of BAP1 (F, ×200). BAP1, BRCA1‐associated protein 1; CEA, carcinoembryonic antigen; EMA, epithelial membrane antigen

Chest radiography and percutaneous echocardiography revealed massive pericardial effusion, but his cardiac function was normal (Figure 1A,B). Chest computed tomography (CT) and positron emission tomography‐CT showed a small nodular uptake of ^18^F‐fluorodeoxyglucose on the pericardium (Figure 1C–F). He was admitted to our department for pericardial drainage. More than 2000 ml of cardiac effusion was drained and cardiac enlargement improved. However, after 2 months, cardiac enlargement was observed, with the same volume of effusion as that before drainage on radiography. The hyaluronic acid level in the pericardial fluid was 329,000 ng/ml, but cytology findings indicated class III (Figure 2A). Haematoxylin–eosin staining of the cell blocks showed diffuse proliferation of atypical cells with enlarged nuclei and eosinophilic cytoplasm, as well as some papillary structures (Figure 2B). Immunostaining revealed that the tumour cells were diffusely positive for calretinin, D2‐40 and WT‐1; focally positive for epithelial membrane antigen (EMA); and negative for carcinoembryonic antigen (CEA), TTF‐1 and desmin. Additionally, BRCA1‐associated protein 1 (BAP1) loss was also observed (Figure 2C–F). This eventually led to the pathological diagnosis of pericardial mesothelioma.

Pericardial fenestration has a high risk of intrathoracic dissemination. Pericardial adhesions were inefficient because of the large amount of pericardial fluid production and increased risk of constrictive pericarditis. Systemic chemotherapy was difficult because he could not cooperate with treatment owing to dementia. We continued symptomatic treatment with pericardial drainage every 3 months; the patient remains alive with no disease progression for 3 years.

## DISCUSSION

MPM is an extremely rare tumour arising from epicardial and pericardial mesothelial cells, accounting for approximately 0.7% of all mesotheliomas.^1^ The median survival time from the first symptom is less than 6 months, which indicates a poor prognosis.^2^ Pericardial effusion, cardiac tamponade and heart failure are common clinical presentations of MPM.^3^

The diagnosis of MPM is difficult. Pericardial biopsy is difficult to perform in some cases because pericardiectomy confers a high risk of death, and an adequate sample may be unobtainable through CT or ultrasound‐guided biopsies to make a definitive diagnosis. In these cases, immunostaining investigations using a cell block of pericardial effusion collected by pericardiocentesis are effective.

In mesothelioma, positive markers, such as CK5/6, calretinin, WT‐1, HBME‐1, thrombomodulin, mesothelin and D2‐40, are commonly used, and negative markers such as TTF‐1, CEA, MOC31, BG8 and Ber‐EP4 are rarely expressed.^4^, ^5^ The combinations of more than two positive and negative immunohistochemical markers are necessary to make an accurate diagnosis.

Immunocytochemical studies are also useful for distinguishing malignant tumours from benign diseases. The finding of the loss of BAP1 is 100% specific for malignant mesothelioma, and it has not been reported in benign mesothelial proliferation.^6^ Well‐differentiated papillary mesothelioma (WDPM) is also known as the indolent type of mesothelioma, which shows a positive staining result for BAP1.^7^, ^8^ The clinical course of slow progression is atypical for MPM. However, immunostaining results indicate the diagnosis of a malignant mesothelioma rather than WDPM or reactive mesothelioma. Lee et al. reported that WDPM cases with BAP1 loss evolve as diffuse, widespread malignant mesothelioma 10 years after the initial diagnosis, suggesting that BAP1 loss might be a predictor of subsequent development of a malignant mesothelioma.^8^ Because our case may be a process in which a WDPM develops into a malignant mesothelioma, careful follow‐up is needed.

We should also consider the possibility of malignant mesothelioma in situ (MMIS), known as a precursor to invasive mesothelioma. MMIS is a pre‐invasive single layer surface proliferation of neoplastic mesothelial cells, and shows BAP1 loss by immunohistochemistry.^9^ Cytological atypia cannot be used to detect MMIS because MMIS has various degrees of cytologic atypia and some reactive mesothelium may be very atypical. On the other hand, BAP1 loss confers a high risk of subsequent appearance of invasive mesothelioma.^10^ Because pericardial biopsy is difficult, it may not be possible to distinguish between this case and invasive mesothelioma and MMIS. However, Churg et al. showed that MMIS slowly progresses to invasive mesothelioma, with a median time from biopsy of 60 months.^10^ The relatively extended time to progression potentially allows for an early therapeutic intervention before that event occurs. In this case, we should consider the possibility of WDPM or MMIS as the differential diagnosis because the clinical course that the patient survives over 3 years without chemotherapy is atypical for MPM. We should make a clinical decision in each case, weighing the gains of treatment against patient age, performance status and complications of procedures.

We encountered a rare case of MPM diagnosed by immunostaining examination using a cell block of the pericardial fluid, which achieved long‐term survival with periodic pericardial drainage. The finding of BAP1 loss is quite useful in the diagnosis of invasive mesothelioma and MMIS. We should lower the threshold for BAP1 immunostaining in cases of recurrent pericardial effusion for an early diagnosis of MPM.

## AUTHOR CONTRIBUTION

All authors contributed to the patient's therapy and this submission. All authors contributed to the conception and interpretation of the work and drafting and revision of the work.

## CONFLICT OF INTEREST

None declared.

## ETHICS STATEMENT

The authors declare that appropriate written informed consent was obtained from the patient for the publication of this manuscript and any accompanying images.

## Data Availability

The data that support the findings of this study are available on request from the corresponding author. The data are not publicly available due to privacy or ethical restrictions.
